# The Outcome of Arthroscopic Anterior Cruciate Ligament Reconstruction in Low-Demand, Non-athletic Patients Following a Home-Based Rehabilitation Protocol

**DOI:** 10.7759/cureus.39851

**Published:** 2023-06-01

**Authors:** Satyam Singh, Rehan Ul Haq, Jitesh Arora

**Affiliations:** 1 Orthopaedics, Dr. Ram Manohar Lohia Institute of Medical Sciences, Lucknow, IND; 2 Orthopaedics, All India Institute of Medical Sciences, Bhopal, IND

**Keywords:** non-athletes, rehabilitation, ligament reconstruction, arthroscopic, anterior cruciate ligament

## Abstract

Background: Arthroscopic anterior cruciate ligament (ACL) reconstruction is a common orthopedic procedure. Most of the literature is on high-demand athletic patients, with little information about the outcomes of low-demand patients. Therefore, we aim to assess the outcomes of non-athletic patients following home-based rehabilitation.

Methods: An observational cross-sectional comparative study was conducted with 30 non-athletic adults with ACL injuries whose pre-injury Tegner activity level was four or less. After six months of reconstruction, patients were assessed for functional outcomes using the Tegner activity level, Lysholm score, International Knee Documentation Committee (IKDC) score, and ACL quality of life (QOL) score. Functional performance was assessed by the carioca test, one-leg hop test, and shuttle test. Functional outcome and performance were compared with an age, sex, and activity level-matched group. Knee stability was assessed by Lachman, anterior drawer, and pivot shift.

Results: All patients returned to their pre-injury Tegner activity level. A statistically significant difference was seen in the Lysholm score, IKDC score, ACL QOL score, carioca test, shuttle test, and one leg hop test (p= <0.001 in each); >5mm of translation of the tibia in the Lachman test was seen in three patients, whereas one patient had >5 mm of translation in the anterior drawer test but pivot shift was absent in all.

Conclusion: We found that all patients returned to their pre-injury Tegner activity level. Most patients had improved knee stability; however, functional outcomes and performance were lower compared to the control group. Therefore, arthroscopic ACL reconstruction is a reasonable treatment option for non-athletic, low-demand patients to get back to their pre-injury functional activity level.

## Introduction

The anterior cruciate ligament (ACL) is the most commonly injured ligament in the knee [1,2]. The incidence of isolated ACL tears is estimated to be 30 per 100,000 people per year in the Western population [3]. The incidence of ACL tears in the Indian population is not well-established in the literature. ACL injuries in athletes are managed with ACL reconstruction without any doubt, and there is a huge body of evidence supporting the need for ACL reconstruction in athletes. However, there are wide differences of opinion on managing ACL injuries in non-athletes [4]. Low-demand patients with ACL injuries who are managed traditionally with conservative methods have a high incidence of symptomatic instability, meniscal, and cartilage injuries, and thus early degenerative osteoarthritis sets in [4]. Most of the patients we treat sustain ACL injuries following road traffic accidents or falls from stairs. Many of these patients are low-demand, non-athletes by profession, and an ACL injury in them does not allow them to carry out their daily living activities like climbing stairs, getting in and out of public transport, and walking barefoot on uneven ground. There is no clear consensus on the indications for surgery for low-demand patients.

With accelerated rehabilitation becoming the standard of care after ACL reconstruction in the past two decades, there has been a trend to rely on physiotherapists to achieve the best results. Very few studies have shown that home-based rehabilitation has a similar outcome as compared to supervised rehabilitation programs [5-9]. However, due to limited physiotherapy facilities, our patients usually go on for goal-directed home-based rehabilitation. Therefore, the aim of the present study is to assess the outcome of arthroscopic ACL reconstruction in non-athletic, low-demand patients following a home-based rehabilitation protocol.

## Materials and methods

This observational cross-sectional comparative study was carried out at the University College of Medical Sciences, New Delhi, India, between November 2016 and April 2018. Written informed consent was obtained from the patients, and ethical clearance was obtained from the ethical committee of the institution. Thirty non-athletic adults (28 men and two women with a mean age group of 28.13 ± 7.15) with a Tegner activity level of four or less who have undergone arthroscopic ACL reconstruction using quadruple hamstring grafts were included in the study (Tegner activity level 1: n=1, level 2: n=9, level 3: n=13, and level 4: n=7). Patients with multiple ligamentous injuries of the knee, contralateral knees with ligament injuries, knees with pre-existing arthritic changes, and polytrauma patients were excluded. Every patient was matched with a participant chosen based on age, sex, and activity who was ± two years older and had a Tegner activity level of ±1 as compared to the patient.

Thirty adults, 28 men and two women, with a mean age group of 27.33 ± 6.5 were included in the activity-matched control group (Tegner activity level 1: n n=0, level 2: n=10, level 3: n=10, level 4: n=7, and level 5: n=3).

All patients with symptomatic ACL injuries underwent arthroscopic transportal reconstruction using a quadruple hamstring graft. Following their discharge, these patients underwent a home-based rehabilitation program (Table 1).

**Table 1:** Home-based rehabilitation protocol following ACL reconstruction

Goals in 0 to two weeks	Modes of achieving these goals
Pain reduction	Analgesics
Swelling reduction	Ice fomentation and compression
Flexion of the knee up to 90 degrees and full extension	Early mobilization of the patient with the help of two axillary crutches and a knee brace
Weight-bearing as tolerated by the patient with the help of two axillary crutches and a knee brace	Static quadriceps and hamstring exercises
Quadriceps and hamstring development	Closed-chain knee flexion and static extension
Goals in two to six weeks	Modes of achieving these goals
Patient free from all medicines	Static quadriceps and hamstring exercises
Full range of motion by the end of six weeks	Axillary crutches to be discarded
Walk with a knee brace without the support of axillary crutches	Prone hangs and passive full extension of the knee
Quadriceps and hamstring development	Half squats
Goals in six weeks to three months	Modes of achieving these goals
Comfortable full range of motion without pain	Slow-form running
Return to pre-injury activity at the end of three months	Wall slides
Quadriceps and hamstring development	Single leg squats
Brace removal	Static cycling
	Return to pre-injury activities except those that involve pivoting
Goals in three to six months	Modes of achieving these goals
Good quadriceps and hamstring muscle strength	Agility training
Satisfactory clinical examination	Maintenance exercises

At the completion of six months of follow-up, the patients were evaluated for functional outcome with Tegner activity level, Lysholm score, International Knee Documentation Committee (IKDC) score, and ACL quality of life score [10].

Functional performance was assessed using the below tests:

Carioca test: The patient was made to run laterally, first towards the right side and then towards the left side, for a distance of 40 feet in each direction, and the total time required was recorded [11].

One-leg hop test: The patient was asked to stand on the injured leg and jump as far forward as possible three times. The longest distance of the jump was used for analysis [11].

Shuttle test: The patient was asked to run four lengths back and forth for 20 feet. The total time required to cover a distance of 80 feet with three changes in direction was recorded [11].

The functional outcome and functional performance were compared with those of the age, sex, and activity-matched group.

Tests to assess the stability of the knee joint:

Anterior drawer test [12]: According to the IKDC, the anterior drawer test was rated as normal (0 mm to 2 mm), nearly normal (3 mm to 5 mm), abnormal (6 mm to 10 mm), and severely abnormal (>10 mm) based on the amount of anterior tibial translation on the injured side compared to the uninjured knee.

Lachman test [13]: As described in IKDC 2000, it was graded as normal (-1 mm to 2 mm), nearly normal (3 mm to 5 mm), abnormal (6 mm to 10 mm), and severely abnormal (>10 mm) based on the amount of tibial translation on the injured side as compared to the uninjured side.

Pivot shift test [14]: According to IKDC 2000, it is graded as equal, glide (+), clunk (+), and gross (+++).

Thigh girth was measured at 15 cm above the medial joint line. The knee stability and the thigh girth were compared with the contralateral limb.

## Results

All the patients returned to their pre-injury Tegner activity level, and it was found that no patient had deterioration of the Tegner activity level. The mean values of the Lysholm score, IKDC score, and ACL QOL score in the patient group and in the control group are shown in Table 2, and it was found that there was a statistically significant difference in all three scores between the two groups. The carioca test and shuttle test were performed by all the patients, and their mean values are shown in Table 2. 

**Table 2:** Results of the anterior drawer test and the Lachmann test in case and control groups

	Case (n=30)		Control (n=30)			
	Anterior drawer test (n)	Lachmann test (n)	Anterior drawer test (n)	Lachmann test (n)	p-value for the anterior drawer test	p-value for the Lachmann test
< 2mm	7	7	30	30	<0.001	<0.001
3 - 5mm	22	20	0	0	<0.001	<0.001
5 - 10mm	1	3	0	0	0.157	0.038
>10mm	0	0	0	0	-	-
TOTAL	30	30	30	30		

A significant difference was noted for both tests (p-value <0.001) between the two groups. One leg hop test was not attempted by two patients, and the mean value was 109.39 ± 16.92 cm in the patient group and 129.63 ± 13.38 cm in the control group (p-value <0.001). The mean value of thigh girth in the involved limb was 41.02 ± 4.47, and in the uninvolved limb, it was 43.60 ± 3.96. Nineteen patients had thigh girths of two or less than two cm when compared with the opposite limb. There was a statistically significant difference observed between the involved and uninvolved limbs (p<0.001).On performing the anterior drawer test, seven patients had a translation of the tibia of < 2 mm, 22 patients had a translation of 3 mm to 5 mm, only one patient had a translation of 5 mm to 10 mm, and no patient had a translation of more than 10 mm, whereas, in the control group, all 30 participants had a translation of < 2 mm (Table 3). On performing the Lachman test, seven patients had a translation of the tibia of < 2 mm, 20 patients had a translation of 3 mm to 5 mm, three patients had a translation of 5 mm to 10 mm, and no patient had a translation of more than 10 mm. In the control group, all 30 participants had a translation of < 2 mm (Table 3). The pivot shift test was absent in nine patients, the glide of the tibia was present in 21 patients, and no patient had clunk or gross translation, whereas, in the control group, all 30 participants had an absent pivot shift test (p=<0.001).

**Table 3:** Outcome of various parameters among patients and controls IKDC: International Knee Documentation Committee; ACL QOL: anterior cruciate ligament quality of life

	Case (n=30)	Control (n=30)	p-value
	Mean ± SD	Mean ± SD	
Lysholm score	84.77±9.7	97.40±3.5	<0.001
IKDC score	76.39±9.18	97.01±4.46	<0.001
ACL QOL Score	79.24±12.35	96.37±5.13	<0.001
Carioca test	64.57±25.09	46.03±3.87	<0.001
Shuttle test	17.53±4.94	13.77±2.25	<0.001

## Discussion

All patients participating in the study, although non-athletic, complained of instability, episodes of knee pain, and loss of pivoting of the lower limb in their day-to-day activities and were therefore considered candidates for surgery. Most of the literature supports the need for ACL reconstruction in high-demand knees, like in athletes, whereas there is no clear consensus on the indications for surgery in low-demand non-athletes.

We found that all the patients in the study were able to return to their pre-injury Tegner activity level at the final follow-up. However, both the functional outcome as measured by the Lysholm score, IKDC score, and ACL QOL score and the functional performance as assessed by the one-leg hop test, carioca test, and shuttle test were significantly lower when compared to an age, sex, and activity-matched control group. The patient's thigh girth was also less as compared to the contralateral limb. More than 90% of patients had a stable knee as assessed by the anterior drawer test, Lachman test, and pivot shift test. None of the patients reported difficulty pivoting or instability of the knee. However, in spite of the knee being stable as assessed by the tests of stability, patients were apprehensive about the status of their knee and therefore not very comfortable exposing their knee to tests like one leg hop for distance (the test was not attempted by two patients), which involved jumping to the farthest possible distance on the affected knee. The carioca test involved repeated twisting activity of the knee, and the shuttle test involved sudden acceleration and deceleration along with a sudden change in direction. Therefore, we feel that a more robust and goal-oriented program with additional preoperative rehabilitation sessions can probably be included in the home-based program already existing in our hospital. The addition of neuromuscular and proprioception training may also be useful. Moreover, patients need to be reminded of the importance of postoperative exercises and motivated to do the same during every clinical visit.

There are very few studies in the literature that support the need for ACL reconstruction in non-athletes. In a retrospective Indian study done by Joseph C., Pathak SS, and Aravinda M. [4] in 2008 (n=1375), a comparison was made between the incidence of associated injuries in the athletes and non-athletes groups. There was a statistically significant increase in the incidence of meniscal injuries and cartilage injuries after one year in both groups. There was no difference in the incidence of meniscal and cartilage injuries in athletes and non-athletes among the corresponding groups. These results demonstrate that both athletes and non-athletes are equally susceptible to long-term meniscal and cartilage injuries if ACL reconstruction is not carried out early. Hence, it was concluded that there should not be any difference in treating low-demand non-athletes as compared to athletes.

The traditional idea of conservatively treating ACL injuries in non-athletes is supported by many authors. A prospective study by Kumar S., Singh K., and Chadha G. [15] with 132 patients, of whom 68 were treated surgically (arthroscopic ACL reconstruction and rehabilitation) and 64 were treated conservatively (optional debridement and rehabilitation). The outcomes were compared using the anterior laxity of the knee joint (by using Lachman’s and pivot shift tests), the Knee Injury and Osteoarthritis Outcome Score (KOOS), the International Knee Documentation Committee (IKDC) score, and the Tegner Activity Level (TAL). This study revealed both the conservatively treated and the reconstructed groups performed similarly, except for a higher objectively measurable instability in the conservative group. They observed no difference in activity level or functional outcome among the two groups on final follow-up. Minimal clinically important differences (MCID) are patient-derived scores that reflect changes in a clinical intervention that are meaningful for the patient. MCID is difficult to define using a subjective patient-based outcome measure like the Lysholm score, the IKDC score, or the ACL QOL score. Moreover, there is a general lack of consensus on how to calculate this difference. In our study, the statistically significant difference observed in the patient-based outcome measures between the patients and the control group did not translate itself into any deterioration in the Tegner activity scale level. Also, the majority of patients, if graded according to the Lysholm score, had a fair to excellent result, with no patient having a poor score.

Rehabilitation following ACL reconstruction is an important component of the treatment of ACL injuries. Controversy exists about the ideal rehabilitation protocol after ACL reconstruction. Therefore, it is important to investigate what level of supervised rehabilitation is required for the patient to attain a good outcome, especially for non-athletic patients whose main target is to return to their pre-injury activity level. With accelerated rehabilitation becoming the standard of care after ACL reconstruction, there has been an overreliance on physical therapists and trainers to motivate, educate, and monitor these patients. However, the literature has shown similar results for home-based rehabilitation when compared to supervised physiotherapy. This study focuses on assessing the outcome of arthroscopic ACL reconstruction in non-athletic patients using a home-based rehabilitation protocol, thereby helping to understand whether unsupervised rehabilitation can lead to a good outcome in this group of patients. As in our study, the good results of a home-based rehabilitation protocol following ACL reconstruction are also supported by many authors [5-9].

In this study, we recommend that ACL reconstruction be considered a primary option for non-athlete, low-demand patients in order to help them get back to their pre-injury activity level, prevent secondary meniscal and cartilage injuries, and thus delay the onset of degenerative osteoarthritis. However, a long-term randomized control trial is to be conducted to confirm the need for ACL reconstruction in the non-athlete population.

The strength of our study is that the outcome of ACL reconstruction has been studied in non-athletic patients who have low demands. This group of patients is usually borderline cases for ACL reconstruction; however, as all patients had symptoms of instability, they were considered suitable for ACL reconstruction after an adequate trial of conservative treatment had failed. All patients reported improvement following surgery, demonstrating that ACL reconstruction may be a useful procedure even for low-demand individuals. The limitation of our study is that we did not have a control group to compare the outcome of home-based rehabilitation with any other type of rehabilitation program. An adequately powered, randomized, and controlled trial comparing different rehabilitation programs would have been ideal. However, due to the limitations of study duration and the number of ACL reconstructions done in our institution, it was not possible to do it. To mitigate this weakness, we compared the outcomes with an age, sex, and activity-matched control group. The other weakness of the study was that it was a retrospective study, and therefore, except for the Tegner activity scale, we did not have any other preoperative scores to compare for the same individual.

## Conclusions

We found that all patients could return to their pre-injury Tegner activity level following arthroscopic ACL reconstruction and a home-based rehabilitation protocol. Most patients had improved knee stability; however, functional outcome and functional performance were lower than in the age, sex, and activity-matched control group. Therefore, we conclude that arthroscopic ACL reconstruction is a reasonable treatment option for non-athletic, low-demand patients to get back to their pre-injury functional activity level with a stable knee.
